# Background Parenchymal Enhancement on Breast MRI as a Prognostic Surrogate: Correlation With Breast Cancer Oncotype Dx Score

**DOI:** 10.3389/fonc.2020.595820

**Published:** 2021-02-04

**Authors:** Michelle Zhang, Meredith Sadinski, Dana Haddad, Min Sun Bae, Danny Martinez, Elizabeth A. Morris, Peter Gibbs, Elizabeth J. Sutton

**Affiliations:** ^1^ Department of Radiology, Memorial Sloan Kettering Cancer Center, New York, NY, United States; ^2^ Department of Radiology, McGill University, Montreal, QC, Canada; ^3^ Department of Radiology, Montefiore, New York, NY, United States; ^4^ Department of Radiology, Mediclinic Middle East, Dubai, United Arab Emirates; ^5^ College of Medicine, Mohammed Bin Rashid University of Medicine and Health Sciences, Dubai, United Arab Emirates; ^6^ Department of Radiology, Seoul National University Hospital, Seoul, South Korea

**Keywords:** breast cancer, magnetic resonance imaging, oncotype, risk score, background parenchymal enhancement

## Abstract

**Purpose:**

Breast MRI background parenchymal enhancement (BPE) can potentially serve as a prognostic marker, by possible correlation with molecular subtype. Oncotype Dx, a gene assay, is a prognostic and predictive surrogate for tumor aggressiveness and treatment response. The purpose of this study was to investigate the association between contralateral non-tumor breast magnetic resonance imaging (MRI) background parenchymal enhancement and tumor oncotype score.

**Methods:**

In this retrospective study, patients with ER+ and HER2− early stage invasive ductal carcinoma who underwent preoperative breast MRI, oncotype risk scoring, and breast conservation surgery from 2008–2010 were identified. After registration, BPE from the pre and three post-contrast phases was automatically extracted using a k-means clustering algorithm. Four metrics were calculated: initial enhancement (IE) relative to the pre-contrast signal, late enhancement, overall enhancement (OE), and area under the enhancement curve (AUC). Histogram analysis was performed to determine first order metrics which were compared to oncotype risk score groups using Mann–Whitney tests and Spearman rank correlation analysis.

**Results:**

This study included 80 women (mean age = 51.1 ± 10.3 years); 46 women were categorized as low risk (≤17) and 34 women were categorized as intermediate/high risk (≥18) according to Oncotype Dx. For the mean of the top 10% pixels, significant differences were noted for IE (p = 0.032), OE (p = 0.049), and AUC (p = 0.044). Using the risk score as a continuous variable, correlation analysis revealed a weak but significant correlation with the mean of the top 10% pixels for IE (r = 0.26, p = 0.02), OE (r = 0.25, p = 0.02), and AUC (r = 0.27, p = 0.02).

**Conclusion:**

BPE metrics of enhancement in the non-tumor breast are associated with tumor Oncotype Dx recurrence score, suggesting that the breast microenvironment may relate to likelihood of recurrence and magnitude of chemotherapy benefit.

## Introduction

According to the Breast Imaging Reporting and Data System (BI-RADS) guidelines, background parenchymal enhancement (BPE) should be routinely reported for breast magnetic resonance imaging (MRI). BPE can influence tumor detection on MRI and the sensitivity/specificity of the interpretation (1–3). However, there has been mounting evidence that BPE may also be of additional value. Theoretically, it can be a surrogate for the breast vascular microenvironment, with the potential to encourage or discourage tumor development, growth, and response to treatment. Higher BPE is associated with high risk patients (such as BRCA carriers) and premenopausal hormonal status (2). Recent preliminary studies suggest that BPE may serve as a prognostic marker, with findings supporting a possible correlation between BPE and molecular subtypes, particularly for distinguishing between luminal A and luminal B type cancers, and a possible relationship with recurrence-free survival (4–6). Although BPE is at times a relatively subjective BI-RADS descriptor, multiple papers have attempted to quantify BPE objectively, finding that it is positively correlated with breast cancer odds, even after adjusting for the amount of fibroglandular tissue (FGT) (7, 8).

Oncotype Dx (Genomic Health Inc., Redwood City, CA) is a validated 21-gene assay which is both prognostic and predictive. It provides the 10-year likelihood of breast cancer recurrence and helps to predict the likelihood of benefit from chemotherapy in patients diagnosed with early-stage estrogen receptor positive (ER+) and human epidermal growth factor receptor 2 negative (HER2−) breast cancer. The Oncotype Dx Recurrence Score (ODxRS) ranges from 0–100 and is often subdivided into three risk categories: low (≤17), intermediate (18–30) and high (≥31). Clinically, a low ODxRS can potentially change patient management, because chemotherapy may not be recommended due to low benefits. Studies have validated the Oncotype Dx test in both node negative and positive patients (9, 10). Currently, ODxRS is being incorporated into breast cancer treatment guidelines (11).

The tumor microenvironment is a complex entity with both intrinsic (e.g., DNA abnormality) and extrinsic characteristics (e.g., oxygen tension and nutrients) (12). The enhancement of the breast is related to its vascularity, and it would be logical to hypothesize that the blood flow to the breast, which brings in the necessary nutrients, oxygen and other metabolites, is contributing to the metabolic environment with certain factors that could promote or discourage tumoral cellular growth. In fact, it has been shown that there is significant correlation between BPE and breast parenchymal metabolic activity as measured by 2-deoxy-2-[fluorine-18]fluoro-D-glucose positron emission tomography/computed tomography (18F-FDG PET/CT) (13, 14). Thus, the increased metabolic activity of a breast with increased BPE could potentially provide a more favorable environment for tumoral growth and be a marker for breast cancer aggressiveness. In addition, BPE can be influenced by both endogenous and exogenous hormones; for example, it has been shown to be sensitive to the menstrual cycle fluctuations (15). Increase in hormonal stimulation such as during breastfeeding or ingestion of hormonal replacement therapy (HRT) has been shown to increase BPE, whereas hormonal suppression therapy such as aromatase inhibitors and Tamoxifen has been shown to decrease BPE (15, 16). By extension, as hormonal variability confers changes to the BPE, it could also in turn confer breast cancer risk stratification.

On MRI, a patient’s breasts usually demonstrate symmetric parenchymal enhancement with similar kinetic curves throughout the contrast-enhanced time course, consistent with their having similar microenvironments. Because of this, we hypothesize that in women with breast cancer, the contralateral healthy breast represents the microenvironment from which the malignancy developed and could be predictive of tumor aggressiveness. The purpose of this study was to investigate if there is a relationship between breast cancer ODxRS and BPE in the contralateral healthy breast. This may elucidate the influence of BPE on tumor behavior and response to treatment using an objective metric measurement of BPE.

## Materials and Methods

### Patients

The institutional review board approved this Health Insurance Portability and Accountability Act-compliant retrospective study and waived written informed consent. Between 2008 and 2010, we identified 80 consecutive breast cancer patients with ER+ and HER2− early stage invasive ductal carcinoma who underwent preoperative breast MRI, Oncotype Dx and breast conservation surgery at our institution. Early stage cancer was defined as TMN staging 1 or 2. Patients with a prior history of breast cancer were excluded. All 80/80 patients overlap with the cohort used by Sutton et al. in 2015 (17). The prior study evaluated the associated between Oncotype Dx and the morphologic and texture-based image features of the cancer on MRI, whereas here we investigated the association between the contralateral non-tumor breast MRI FGT enhancement and tumor oncotype score.

### Imaging Protocol

Breast MRI was performed using a protocol which included a pre-contrast T1-weighted sequence and three T1-weighted post-contrast phases. All images were acquired with a 1.5T (n = 47; 59%) or 3.0T (n = 33; 41%) MRI system (Signa or Signa HDX; GE Medical Systems, Waukesha, WI). A dedicated 8 channel breast coil was used in all patients. Sagittal T1-weighted fat-suppressed 2D multi-slice acquisitions were acquired before and sequentially three times after intravenous administration of 0.1 mmol gadopentetate dimeglumine per kilogram body weight (Magnevist; Berlex Laboratories/Bayer Health Care Pharmaceuticals, Montville, NJ) at a rate of 2 ml/sec with an automatic injector (Medrad, Warrendale, PA) and a 20-second scan delay using the following parameters: repetition time (TR, sec)/echo time (TE, sec), 6.81 (5.08–11.30)/4.20 (1.95–4.20); flip angle (°), 10 (10–12), acquisition matrix, 256×192 (256–320×160–256); in-plane resolution (mm), 0.78×0.78 (0.39–0.94); slice thickness (mm), 3 (3–3); and temporal resolution ~90 secs.

### Imaging Analysis

BPE of the contralateral non-cancerous breast was analyzed on a pixel-by-pixel basis. The fibroglandular tissue was segmented semi-automatically as follows using custom software written in MATLAB (Mathworks, Natick, NA). A radiologist (MZ, radiologist with breast imaging fellowship) first drew a line segment separating the chest wall from the breast tissue on orthogonal sagittal and axial maximum intensity projections of the first post-contrast T1-weighted image. A Gaussian smoothing filter was then applied, and the breast tissue was automatically thresholded from the background. An erosion filter was applied to the resulting segmentation to compensate for overestimation due to the smoothing step and to exclude skin. A rigid, intensity-based registration was applied to the pre- and post-contrast images to account for patient motion during the exam. The breast segmentation was then propagated to the pre-contrast T1-weighted image and the fibroglandular tissue was extracted using k-means clustering on the pre-contrast T1-weighted image. Finally, image registration and segmentation were checked by another radiologist (DH, radiologist with breast imaging fellowship), with manually adjustment applied as necessary.

**Figure 1:**
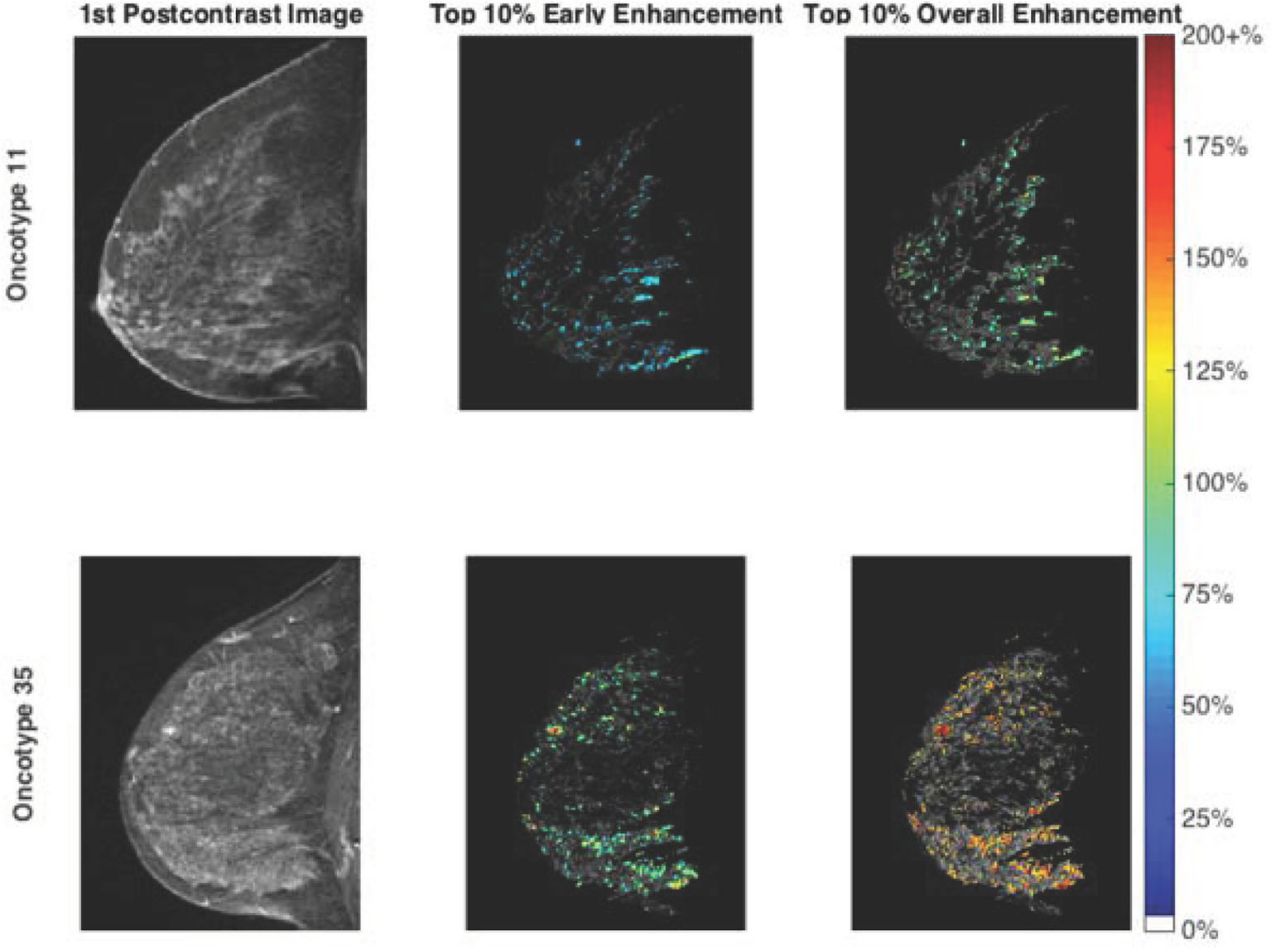
Fibroglandular tissue enhancement in a low risk group patient (top row) and a high risk group patient (bottom row). The first postcontrast images are illustrated (left) alongside segmented fibroglandular tissue with early enhancement overlay (middle) and overall enhancement overlay (right). Note the higher enhancement values evident for the high risk group patient. Oncotype Dx is a prognostic and predictive surrogate for tumor aggressiveness and treatment response. A Oncotype Dx score of 11 is a representative example of a patient that is a low risk. Oncotype Dx score of 35 is a representative example of a patient that is at intermediate/high risk. The “top 10%” corresponds to the voxels exhibiting the 10% highest enhancement level in the middle and right figures.

Initial, overall, and late enhancement were calculated using the pre-contrast T1-weighted signal as the baseline. Initial enhancement (IE) was calculated as the percentage increase in signal from the pre-contrast (baseline) image to the first post-contrast image with respect to the baseline signal. Similarly, overall enhancement (OE) was calculated as the percentage increase in signal from baseline to the final post-contrast image with respect to the baseline signal. Late enhancement was evaluated as the percentage increase in signal from the first post contrast image to the last post contrast image with respect to the first post contrast image. Finally, the area under the enhancement curve (AUC) for total contrast enhancement was calculated, normalized to the baseline signal intensity (**Figure 1**).

All pixels with negative signal changes post-contrast injection were removed, since this indicates that no enhancement has occurred, and any signal fluctuations noted are due to signal to noise effects. Similarly, pixels with enhancement over 300% were removed and regarded as demonstrating non-physiologically acceptable BPE. Such high percentage enhancement rates can be attributed to low pre-contrast signal followed by normal signal to noise-based fluctuations in the signal recorded during the dynamic time course.

Objective BPE calculations were presented as median and interquartile ranges. Since negative values of initial and total enhancement are precluded by definition, the pixel-by-pixel histogram of enhancement for each individual case could be considered as non-normally distributed. Therefore, median values and percentiles were used as summary variables for the central tendency and spread of the data. Reflecting previous work by van der Velden et al., the mean of the top 10% of pixels on the enhancement histograms was also calculated for each case, the decision of which was determined *a priori* (18). Focusing on the most enhancing pixels in breast lesions using similar techniques such as hot spot analysis has been shown to provide greater discrimination to predict response to neoadjuvant chemotherapy among patients with breast cancer (19).

### Tissue Testing

All breast cancers underwent the standard Oncotype DX test to derive the Oncotype Dx recurrence score (ODxRS). Using ODxRS, patients were dichotomized into a low risk group (those with scores ≤17) and an intermediate/high risk group (those with scores ≥18). This was deemed appropriate due to the dichotomy of clinical management based on risk stratification by ODxRS; “low risk” patients would potentially not be offered additional chemotherapy (due to low benefit) and the “intermediate/high risk patients” would potentially be offered more aggressive therapies due to higher predicted recurrence and thus higher benefit of chemotherapy.

### Statistical Analysis

Patient and clinical characteristics were compared between patients in the low and intermediate/high groups, using Fisher’s exact test or the chi-square test where appropriate. All analyses were performed using MATLAB (v 9.1.0 R2016b MathWorks, Natick, MA). After appropriate dichotomization comparison of low and intermediate/high risk group, first order statistics derived from the four enhancement curve parameters was performed using the independent sample Mann–Whitney U test, with a p-value < 0.05 regarded as indicating a significant difference between groups. The Spearman rank correlation analysis was used to assess the relationships between the four enhancement curve parameters and the continuous ODxRS metric, with a p-value < 0.05 indicating the presence of a significant correlation between parameters.

## Results

### Patients

The mean age of the study population was 51.2 years (range, 27.4–77.6). **Table 1** shows the patient characteristics. All patients underwent breast conserving surgery. The median ODxRS in the study population was 16.5 (range: 0–78). Based on OdxRS, 46 (58%) patients were categorized into the low risk group (0–17) and 34 (42%) patients were categorized into the intermediate/high risk groups (≥18). From **Table 1**, it can be seen that patients in the intermediate/high risk group were more likely to be PR negative (p = 0.028), HER2 positive (p = 0.029), have a higher nuclear grade (p = 0.030), and also less likely to be receiving hormone therapy (p = 0.004), compared with the low risk group. No significant difference in overall BPE score was noted between the two groups (p = 0.642).

**Table 1:** Patient Characteristics.

Categorical variable	Low Risk (n = 46)	Intermediate/High Risk (n = 34)	p-value
**ER status**			0.073
Negative	0 (0%)	3 (9%)	
Positive	46 (100%)	31 (91%)	
**PR status**			0.028
Negative	2 (4%)	7 (21%)	
Positive	44 (96%)	27 (79%)	
**HER2 status**			0.029
Negative	46 (100%)	30 (88%)	
Positive	0 (0%)	4 (12%)	
**Nodal status**			0.464
Negative	42 (90%)	30 (88%)	
Positive	4 (10%)	4 (12%)	
**Histology grade**			0.216
1	4 (9%)	1 (3%)	
2	18 (39%)	9 (26%)	
3	24 (52%)	24 (71%)	
**Nuclear grade**			0.030
1	4 (9%)	0 (0%)	
2	29 (63%)	14 (41%)	
3	10 (22%)	14 (41%)	
Unknown	3 (6%)	6 (18%)	
**Family history**			0.192
No	26 (57%)	15 (44%)	
Yes	20 (43%)	19 (56%)	
Menopausal status			0.207
Pre-menopausal	31 (67%)	19 (56%)	
Post-menopausal	15 (33%)	15 (44%)	
**HRT**			0.106
No	43 (93%)	27 (79%)	
Yes	3 (7%)	6 (18%)	
Unknown	0 (0%)	1 (3%)	
**Hormone therapy**			0.004
No	0 (0%)	6 (18%)	
Yes	46 (100%)	28 (82%)	
**Radiation therapy**			0.203
No	3 (7%)	5 (15%)	
Yes	43 (93%)	29 (85%)	
**BPE**			0.642
Minimal	8 (17%)	7 (21%)	
Mild	17 (37%)	12 (35%)	
Moderate	15 (33%)	7 (21%)	
Marked	6 (13%)	8 (23%)	

HRT, hormone replacement therapy.

### Relationship Between the Four Enhancement Curve Parameters and ODxRS Categories

Borderline significant differences were noted between the two groups using the Mann–Whitney U test for median values of IE (p = 0.07) and OE (p = 0.09). When mean values of the top 10% of pixels were considered, significant differences were noted for IE (p = 0.03), OE (p = 0.049) and AUC (p = 0.04). Greater IE, OE, and AUC were noted for the intermediate/high risk group compared to the low risk group (**Table 2**). **Figure 1** illustrates the first postcontrast images alongside segmented FGT with higher enhancement values for a high risk group patient compared with a low risk group patient.

**Table 2:** Top 10% BPE correlating with dichotomized Oncotype Dx score. Values are given as median (range).

	Initial Enh (%)	Overall Enh (%)	Late Enh (%)	AUC
**Group 1** **(*n* = 46)**	53.1 (20.2 to 129.2)	72.6 (29.9 to 185.0)	26.5 (13.2 to 84.2)	2.71 (1.10 to 6.79)
**Group 2/3** **(*n* = 34)**	63.9 (30.3 to 159.1)	84.5 (53.9 to 195.0)	28.9 (17.9 to 50.5)	3.23 (1.84 to 7.57)
***P*-value**	*0.03*	*0.049*	*0.45*	*0.04*

Enh, Enhancement; AUC, Area under the enhancement curve.

Group 1 = low risk oncotype score group (ODxRS ≤ 17).

Group 2/3 = intermediate/high risk oncotype score group (ODxRS ≥ 18).

### Relationship Between the Four Enhancement Curve Parameters and ODxRS as a Continuous Variable

**Figure 2:**
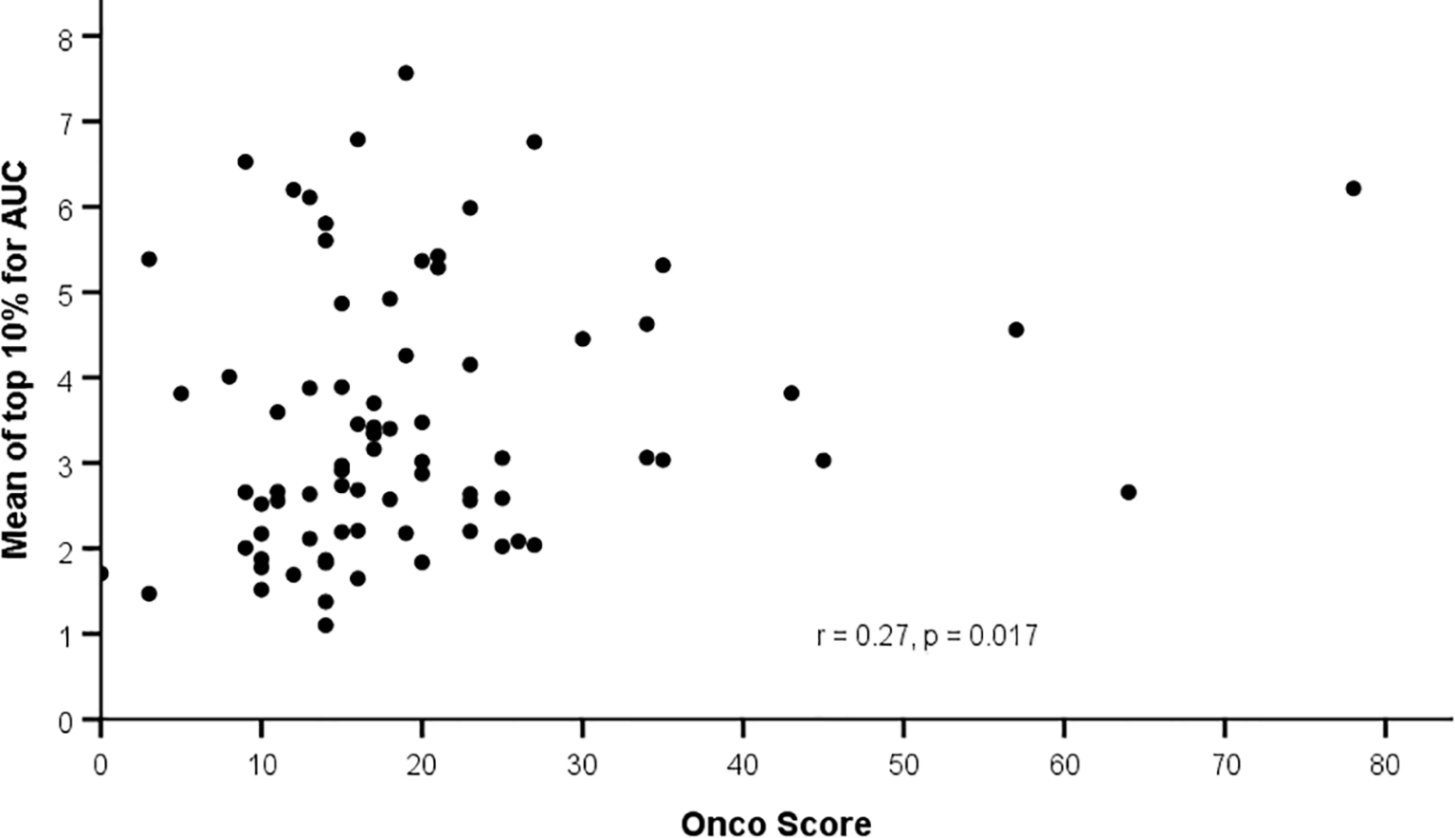
Correlation between background parenchymal enhancement (mean top 10%) and Oncotype Dx Recurrence Score.

Using ODxRS as a continuous variable, Spearman rank correlation analysis revealed weak but significant positive correlations between ODxRS and the mean of the top 10% of pixels for IE (r = 0.26, p = 0.02), OE (r = 0.25, p = 0.02), and AUC (r = 0.27, p = 0.02) (**Figure 2**). No significant correlations were noted when utilizing median values (**Table 3**). With a sample size of 80 and employing a type I error rate of 0.05, then for a correlation of ~0.25 a type II error rate of 0.35 (65% power) can be expected.

**Table 3:** Correlation analysis of BPE metrics with continuous Oncotype Dx score.

	Spearman rank correlation coefficient	*P*-value
**Median**		
**Initial enhancement**	0.20	0.07
**Overall enhancement**	0.22	0.05
**Late enhancement**	0.17	0.13
**AUC**	0.21	0.06
**Top 10% of pixels**		
**Initial enhancement**	0.26	*0.02*
**Overall enhancement**	0.25	*0.02*
**Late enhancement**	0.14	0.22
**AUC**	0.27	*0.02*

BPE, Background parenchymal enhancement; AUC, Area under the enhancement curve.

## Discussion

BPE in the contralateral non-tumor breast positively correlates with ODxRS. This suggests that the microenvironment of the breast may predict tumor biology including the likelihood of recurrence and the magnitude of chemotherapy benefit. Given that we used the quantitative metric measurement of BPE rather than the subjective categorization by the interpreting radiologist, the relationship is more objective and reproducible. The correlation is more significant when considering the top 10% BPE and after dichotomizing ODxRS.

The decision to use the mean of the top 10% of the BPE was determined a *priori*. This is similar to hot spot analysis and was previously proposed by other papers (18, 20). Given that we hypothesized that the BPE represents the microenvironment from which the tumor arose, the top enhancing portion of the breast is mostly likely the most hormonally active, or susceptible. While there may be great heterogeneity across the breast in terms of BPE, focusing on the top 10% would ensure honing in on the most vascularized and sensitive part of the breast. This was affirmed by our study which showed a positive correlation between tumor aggressiveness (ODxRS) and the top 10% BPE. In addition, in our study, there was no spatial localization of the top 10% most enhancing parenchyma and the pixels were equally likely to be scattered disparately throughout the breast; therefore, we hypothesize that the risk assessment conveyed *via* BPE would be globally for the breast itself and not for a particular region of the breast.

Interestingly, the correlation between ODxRS and BPE was stronger when the scores are dichotomized rather than when used as a continuous variable. Clinically speaking, low-risk patients are usually not recommended to receive adjuvant chemotherapy whereas intermediate/high-risk patients are recommended to receive additional chemotherapy (21). Thus, this dichotomization would make sense as it would change clinical management. There is substantial benefit gained from additional adjuvant therapy for the high risk group. Management for patients in the intermediate group is somewhat controversial with most patients being offered chemotherapy but the recently published prospective TAILORx trial suggests that endocrine therapy may be non-inferior to chemoendocrine therapy in particular subsets of patients (e.g., older women above 50 years of age with ODxRS score of 25 or lower); however, more validation studies would be needed to better define these subgroups (21–23).

The current literature on the association of BPE with tumor ODxRS is scarce. However, there is growing evidence that the microenvironment of the breast, as evidenced by the BPE, is associated with different tumoral behavior and can provide prognostic factors. It has been shown that BPE in the contralateral breast in breast cancer patients, especially the top 10% parenchymal enhancement as our study has similarly assessed, is associated with overall long-term patient outcome (20). In a similar fashion, increased BPE on pre-treatment MRI for patients undergoing neoadjuvant chemotherapy has been shown to be significantly correlated with worse recurrence-free survival (24). Remarkably, a parallel study looking instead at the BPE around ductal carcinoma *in situ* in the cancerous breast showed that the increased signal enhancement ratio in the ipsilateral cancerous breast around the tumor was associated with worse ipsilateral tumor recurrence-free survival (25).

Our study affirms that breast BPE can serve as a surrogate for the microenvironment that predisposes carcinogenesis, in line with previous studies. It can potentially help predict treatment response and disease recurrence and triage patients into different risk stratification groups. Ultimately, BPE can help to identify tumoral characteristics that would indicate more aggressive behavior and help tailor treatment and clinical decision making. If this association is further validated, additional studies could be performed to evaluate whether this risk would also be conferred in non-cancerous screening patients, in order to help prevent cancer development rather than only passively treating the tumor once it has already developed.

Our study is limited by the fact that it is a retrospective study from a single institution and the sample size was small. In addition, there may also be selection bias as only patients who were stratified by the Oncotype Dx test were included for analysis. Conclusions are limited to the subset of ER+, HER2− breast cancer, and not generalizable to the broader patient population.

In conclusion, increased fibroglandular BPE on MRI in the contralateral non-tumor breast is correlated with higher Oncotype Dx scores, suggesting that BPE may be an imaging phenotype of the microenvironment that correlates with tumor aggressiveness and response to chemotherapy. Future studies with a larger patient sample size and treatment response follow-up may help further elucidate this association. Fibroglandular BPE is a very promising predictive factor as an additional tool for risk stratification and treatment management in patients with breast cancer.

## Data Availability Statement

The data analyzed in this study is subject to the following licenses/restrictions. Data will be shared with qualified researchers whose proposed use of the data has been approved. Requests to access these datasets should be directed to ES (suttone@mskcc.org).

## Ethics Statement

The studies involving human participants were reviewed and approved by Institutional Review Board, Memorial Sloan Kettering Cancer Center. Written informed consent for participation was not required for this study in accordance with the national legislation and the institutional requirements.

## Author Contributions

All authors contributed to the article and approved the submitted version.

## Funding

This research was funded in part through the NIH/NCI Cancer Center Support Grant P30 CA008748, the Breast Cancer Research Foundation, and the Susan G. Komen Foundation.

## Conflict of Interest

EM received a grant from GRAIL Inc. for research not related to the present article. MS is currently employed by Promaxo in San Francisco, CA.

The remaining authors declare that the research was conducted in the absence of any commercial or financial relationships that could be construed as a potential conflict of interest.
